# A droplet acoustofluidic platform for time-controlled microbead-based reactions

**DOI:** 10.1063/5.0050440

**Published:** 2021-05-17

**Authors:** Zhenhua Liu, Anna Fornell, Maria Tenje

**Affiliations:** 1 Department of Materials Science and Engineering, Science for Life Laboratory, Uppsala University, SE-752 37 Uppsala, Sweden; 2 MAX IV Laboratory, Lund University, SE-221 00 Lund, Sweden

## Abstract

Droplet microfluidics is a powerful method used to characterize chemical reactions at high throughput. Often detection is performed via in-line optical readout, which puts high demands on the detection system or makes detection of low concentration substrates challenging. Here, we have developed a droplet acoustofluidic chip for time-controlled reactions that can be combined with off-line optical readout. The principle of the platform is demonstrated by the enzymatic conversion of fluorescein diphosphate to fluorescein by alkaline phosphatase. The novelty of this work is that the time of the enzymatic reaction is controlled by physically removing the enzymes from the droplets instead of using chemical inhibitors. This is advantageous as inhibitors could potentially interact with the readout. Droplets containing substrate were generated on the chip, and enzyme-coupled microbeads were added into the droplets via pico-injection. The reaction starts as soon as the enzyme/bead complexes are added, and the reaction is stopped when the microbeads are removed from the droplets at a channel bifurcation. The encapsulated microbeads were focused in the droplets by acoustophoresis during the split, leaving the product in the side daughter droplet to be collected for the analysis (without beads). The time of the reaction was controlled by using different outlets, positioned at different lengths from the pico-injector. The enzymatic conversion could be measured with fluorescence readout in a separate PDMS based assay chip. We show the ability to perform time-controlled enzymatic assays in droplet microfluidics coupled to an off-line optical readout, without the need of enzyme inhibitors.

## I . INTRODUCTION

Droplet microfluidics is a powerful platform and presents valuable applications in multiple fields.^1^ Compared to continuous-flow microfluidics, droplet microfluidics can reach higher throughput, avoid cross-contamination of sample, and allow for short mixing time and parallel analyses.^2,3^ With the rapid development of microfabrication and microengineering, techniques to achieve droplet internal manipulation or whole droplet manipulation by external fields,^4^ such as dielectrophoresis,^5,6^ acoustophoresis,^7,8^ magnetophoresis,^9,10^ electrocoalescence,^11^ and digital microfluidics^12,13^ have been presented. Droplet microfluidics has presented several key applications as a platform in biological assays,^3,14–16^ including enzymatic reactions and cell culture,^17^ which are usually carried out by continuous-flow microfluidics. Droplet microfluidics has demonstrated an excellent ability in enzymatic kinetic measurements^18–22^ and enzyme inhibition assays.^21,23,24^ The enzymatic reaction occurs when the enzyme solution meets with the substrate solution, and the reaction velocity slows down if an inhibitor is added. To use droplet microfluidics for research on enzymatic kinetics, Sjostrom *et al.* integrated a pico-injector on-chip to achieve high-throughput assays.^21^ They demonstrated the multiplex analysis of enzyme kinetics and inhibition. However, a challenge with this approach was to separate the product from the enzyme since they were mixed together in the same droplet after the injection step. Furthermore, the method required a movable detector to follow the fluorescent intensity of the droplets throughout the whole system. With this approach, it is only possible to evaluate enzymatic reactions generating a sufficiently strong intensity. The issue with the detection limit was addressed by Miller *et al*. who demonstrated a digital microfluidic device for in-droplet enzymatic assays using a plate reader for detection.^18^ They achieved a lower detection limit compared with conventional methods as the platform could allow for longer exposure times. On the other hand, their technology is limited by allowing for only a few droplets to run simultaneously, thus reducing throughput. Here, we show a method to perform time-controlled enzymatic assays in droplet microfluidics by coupling the enzymes via a biotin–streptavidin covalent binding to microbeads which then can be separated from the product of the enzymatic reaction using acoustophoresis. This method allows us to stop the reaction without the need of enzyme inhibitors. The readout is performed off-chip which allows for longer exposure times compared to performing the analysis in-line.

Microbead-based microfluidics is popular in biochemical applications, such as immunoassays,^25^ due to high surface-to-volume ratio of microbeads, their easy transportation into fluid and flexible surface functionalization.^26^ Microbeads in microfluidic channels can be manipulated using techniques such as acoustophoresis and magnetophoresis. Acoustophoresis can be used to control various types of particles, e.g., polystyrene beads, silica beads, poly dimethylsiloxane (PDMS) beads, and cells while magnetophoresis can only be used with magnetic beads. By the combination of droplet microfluidics and microbead-based immunoassays, microbead-based droplet microfluidics has shown excellent abilities for detection and quantitative analysis as a diagnostic tool since it needs less reagents and it can reach high throughput.^27,28^ In this work, we use streptavidin-coated microbeads which are commercially available and offer extremely stable binding with biotinylated molecules, such as antibodies. Furthermore, the microbeads can be easily manipulated in droplets by acoustophoresis.^8^

In this work, we present a droplet acoustofluidic device for microbead-based assays that can be combined with off-chip analysis. To demonstrate the functionality of the platform, we linked the enzyme alkaline phosphatase (ALP) to microbeads via streptavidin–biotin conjugation and the enzymatic reaction was initiated by introducing the enzyme-coupled microbeads into the droplets by pico-injection. The enzymatic reaction was stopped by removing a small part of the droplets at a channel bifurcation under acoustic actuation so that the enzyme-microbeads were collected in the main outlet channel. Here, fluorescein diphosphate (FDP) was chosen as a substrate with the product being fluorescein. The enzymatic reaction time was controlled by the channel length, and the reaction efficacy was determined by measuring the fluorescence intensity in the side daughter droplet (without microbeads) off-chip using fluorescence.

## II. ACOUSTOPHORESIS

Acoustophoresis is a label-free method to manipulate whole droplets in microfluidic channels,^7,29,30^ as well as to sort microbeads encapsulated inside droplets.^8,31–33^ In continuous-flow microfluidics, the primary acoustic radiation force, $F^{\mathrm{rad}}$, in a half wavelength standing wave field is given by^34^

$$F^{\mathrm{rad}}=4\pi \Phi (\tilde{\kappa },\tilde{\rho })ka^{3}E_{\mathrm{ac}}\sin(2ky),$$ (1a)

$$\Phi =\frac{1}{3}\left(\frac{5\tilde{\rho }-2}{2\tilde{\rho }+1}-\tilde{\kappa }\right),$$ (1b)

where *k* is the wavenumber (k=2π/λ and λ is the wavelength), *a* is the particle radius, $E_{\mathrm{ac}}$ is the acoustic energy density, *y* is the distance from the channel wall, Φ is the acoustic contrast factor, $\tilde{\rho }$ is the density ratio between the particle and the surrounding fluid, and $\tilde{\kappa }$ is the compressibility ratio between the particle and the surrounding fluid.

The primary acoustic radiation force causes particles such as cells and plastic microbeads to be focused along the central line of a microfluidic channel at the fundamental resonance frequency. It has previously been shown that to generate a strong and homogeneous standing wave field in two-phase systems, it is important to match the acoustic properties of the continuous phase and the dispersed phase.^35^ Here, light mineral oil was used as a continuous phase as it has similar acoustic properties to water.

## III. METHODS

### A. Design and fabrication of the microfluidic chip

**FIG. 1.**
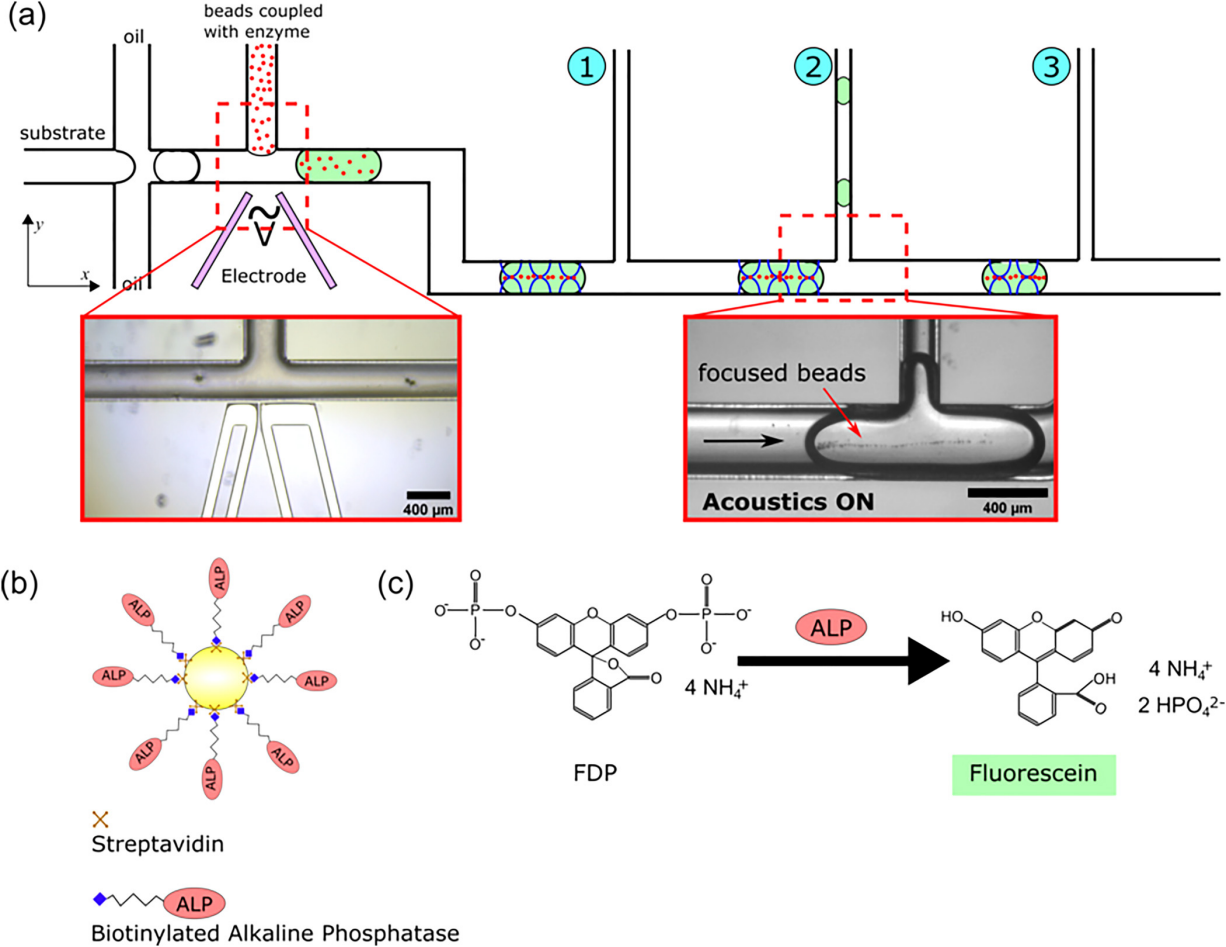
(a) The chip contains droplet generation, pico-injector, and three droplet splits. The 5 *μ*m microbeads can be focused in the center of the droplet by standing bulk acoustic waves. The length of the reaction channel is not according to scale. (b) Schematic drawing of the streptavidin bead coupled with biotinylated ALP. (c) The enzyme reaction, ALP catalyzes FDP to fluorescein.

The main flow circuit of the chip contains droplet generation, pico-injection, a reaction channel, and three droplet splits [Fig. 1(a)]. The channels for the continuous phase have one common inlet. An electrode circuit is integrated on-chip at the pico-injector. The width of the main flow channel is 380 *μ*m, and the height of all channels is 175 *μ*m. The width of the side outlet channels at the droplet splits is 200 *μ*m. The principle of operation is that droplets containing the substrate (FDP) are generated in the microfluidic system, and the reaction is initiated as the enzyme-coupled microbeads are injected into the droplets via the pico-injector. After the droplet split, the beads remain in the main channel and the product can be collected from the side outlet channel for further analysis. The three side outlet channels are placed in series along the main flow channel and they were used independently to split the droplets [Fig. 1(a)]. The length of the reaction channel, which is the channel from the pico-injection to the droplet splits, is either 42, 77, or 112 mm, depending on which droplet split that is active. To achieve good on-chip resonance by standing bulk acoustic waves, the microfluidic channel was fabricated in silicon. The channels were dry-etched on a silicon wafer following standard microfabrication processes as previously reported.^36^ The chip was heated to 100 °C on a hot plate, and a low-temperature solder (No. 158, Indium Corporation) was injected in the electrode channels. A piezoelectric transducer with optimal resonance at 2 MHz (APC-840, Americanpiezo) was glued on the back side of the chip by cyanoacrylate glue (420, Loctite). The fluidic channels were injected with hydrophobic silane (Repel-Silane ES, GE Healthcare) for 1 h and then rinsed with oil. A separate PDMS chip was used to collect the daughter droplets for the analysis ejected from the side outlet channels. The PDMS chip was fabricated by a conventional soft lithography using a SU-8 master. The PDMS chip contains an open chamber, 8 mm in diameter, and 2 mm in depth. There are microwells of 300 *μ*m in diameter and 100 *μ*m in depth on the bottom of the chamber used for trapping the droplets.

### B. Enzymatic reactions

Microbeads were coupled with biotinylated enzyme before pico-injection [Fig. 1(b)]. The streptavidin-coated polystyrene microbeads (5 *μ*m, Bangs Laboratory) were washed three times by phosphate-buffered saline (PBS, ThermoFisher) with 1% bovine serum albumin (Sigma-Aldrich) and then diluted into 1 mg/ml. An aliquot of 20 *μ*l biotinylated ALP (1.7 mg/ml, ThermoFisher) was added into 1 ml diluted bead solution, and the mixture was incubated at room temperature for 1 h with gentle shaking. The beads were washed three times by the same PBS buffer and resuspended in diethanolamine buffer (DEA, ThermoFisher) at a concentration of 0.5 mg/ml. The binding capacity of the streptavidin-coated microbeads with biotin functionalized FITC (fluorescein isothiocyanate) is 0.04 *μ*g/mg according to the certificate of analysis of the product.

FDP (ThermoFisher) was dissolved at a concentration of 10 *μ*M in DEA. The nonfluorescent FDP was converted into fluorescein by catalysis of ALP as shown in Fig. 1(c), and the fluorescein concentration was determined using fluorescence microscopy.

### C. Experimental setup and measurements

**FIG. 2.**
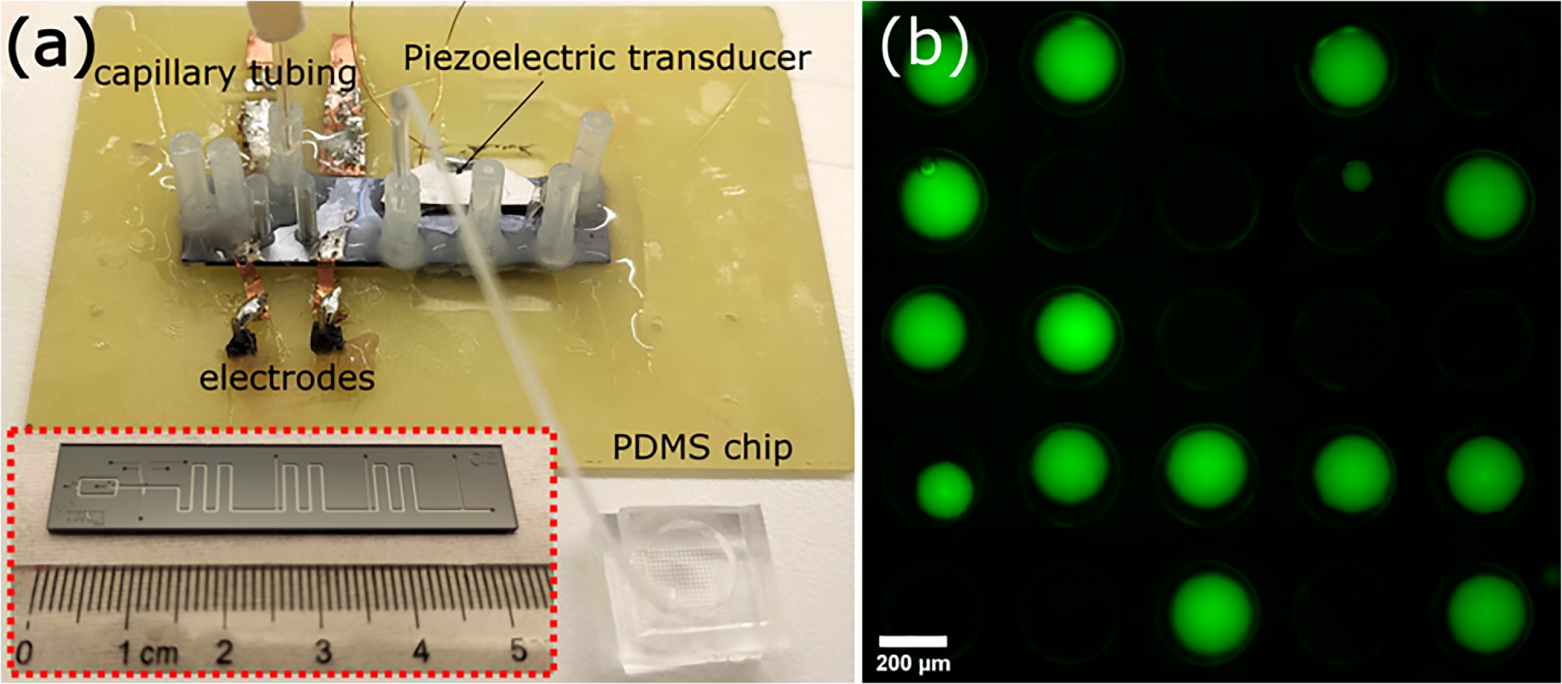
(a) Photograph of the experimental setup. Red inset shows the front-side of the Si/glass droplet acoustofluidic chip. (b) Fluorescence image of the collected droplets in the PDMS chip showing typical loading density in the well array. Only droplets with a diameter >160 *μ*m were used for the analysis.

Light mineral oil (Sigma-Aldrich) with 2% polyglycerol polyricinoleate (PGPR, DuPont) was used as the continuous phase. The dispersed phase was a fluorescein solution (for calibration, Sigma-Aldrich) or FDP solution (for the proof-of-concept study). Glass syringes (1 ml L-Mark, Setonic) were used for injecting fluids, and a plastic syringe (1 ml Syringe Luer-Lok Tip, Becton Dickinson) was connected to the main channel to control the split ratio at each droplet split. A higher withdrawal flow rate causes larger parts of the droplet to remain in the main channel and smaller daughter droplets in side outlet channel. Fluid containing enzyme-coupled microbeads was injected into the pico-injector through glass capillary tubing (Genetec) with an inner diameter of 75 *μ*m to reduce the sedimentation of microbeads in tubing. Other syringes were connected to the chip using polyethylene tubing (Intramedic, Becton Dickinson), and flow rates were controlled by syringe pumps (neMESYS, Cetoni). A 10 cm long piece of tubing (Tygon ND 100-80) with an inner diameter of 0.25 mm was used to transfer the droplets from the side outlet channel to the PDMS chip (Fig. 2).

A total flow rate of 2 *μ*l/min was used for the continuous phase, and a flow rate of 1 *μ*l/min was used for the dispersed phase. The pico-injector was operated at a flow rate of 0.5 *μ*l/min. The reaction time from the pico-injection to the three droplet splits was 45, 81, and 115 s, respectively.

Fluorescein solution (13.3 *μ*M) was used to generate droplets for volume measurements, and images of the droplets on the silicon chip were acquired using a fluorescence microscope (TE2000-U, Nikon) equipped with a camera (DFK NME33UX174, IMAGINGSOURCE). The droplets were then transferred from the silicon chip to the PDMS chip prefilled with light mineral oil with 2% PGPR. As shown in Fig. 2(b), images for the fluorescence measurement of the droplets in the PDMS chip were taken by a digital camera (C11440, Hamamatsu) mounted on a fluorescence microscope (Olympus, IX3). The exposure time was set at 1 s for the fluorescein concentration range between 0.13 and 1.33 *μ*M. Droplets used for the analysis were manually selected, and the intensity was measured using Cell Profiler (Broad Institute).

The electrodes for the pico-injector and the piezoelectric element were connected to a two-channel function generator (AFG3022C, Tektronix). An oscilloscope (TBS 1102B, Tektronix) was used to monitor the applied signals. A sine wave of 1.8 MHz frequency was supplied by the function generator, amplified to 30 V_pp_ by a power amplifier (XPA125B, Xiegu), and then applied over the piezoelectric transducer. A square wave of 5 kHz frequency was supplied by the same function generator, amplified to 100 V_pp_ by another power amplifier (210L, Electronics & Innovation), and then applied on the electrodes for the pico-injection.

### D. Analysis

To measure the droplet volume, images of droplets at different positions on the chip (after droplet generation, after pico-injection, and after the splits) were captured and the droplet volumes were calculated as^37^

$$V=\left[HW-(4-\pi ){\left(\frac{2}{H}+\frac{2}{W}\right)}^{-2}\right]\left(L-\frac{W}{3}\right),$$ (2)

where *H* is the height of the channel, *W* is the width of the channel, and *L* is the length of the droplet.

For the fluorescence intensity measurement, the droplets were transferred to the PDMS chip. The fluorescence intensity was determined using the Cellprofiler software (Broad Institute).^38^ The intensity value was then translated into fluorescein concentration using the calibration curve.

## IV. RESULTS AND DISCUSSIONS

### A. Droplet volume

The volume of 100 droplets was measured after droplet generation, after pico-injection, and after the droplet splits. The total flow rate after pico-injection was 3.5 *μ*l/min. When splitting the droplets in a bifurcation, there is a limit on how large side daughter droplets that can be generated without increased risk of affecting the flow lines inside the droplet with the encapsulated beads so that they are also drawn into the daughter droplet. The split ratio between the main channel and the side outlet channel was adjusted to obtain high bead separation efficiency. In this setup, the droplets had a volume of 48.3 ± 0.7 nl directly after generation and 75.4 ± 0.2 nl after the bead solution had been injected. These values were stable for all different split configurations. It was found that for split 1 (with split 2 and split 3 closed), the optimal withdrawal flow rate was 3.2 *μ*l/min, resulting in 7.3 ± 3.8 nl of the droplet being withdrawn. For both split 2 (with split 1 and split 3 closed) and split 3 (with split 1 and split 2 closed), the optimal withdrawal flow rate was 3 *μ*l/min, resulting in 15.4 ± 4.2 nl droplets and 14.7 ± 4.8 nl side daughter droplets being withdrawn, respectively.

### B. Acoustophoresis

**FIG. 3.**
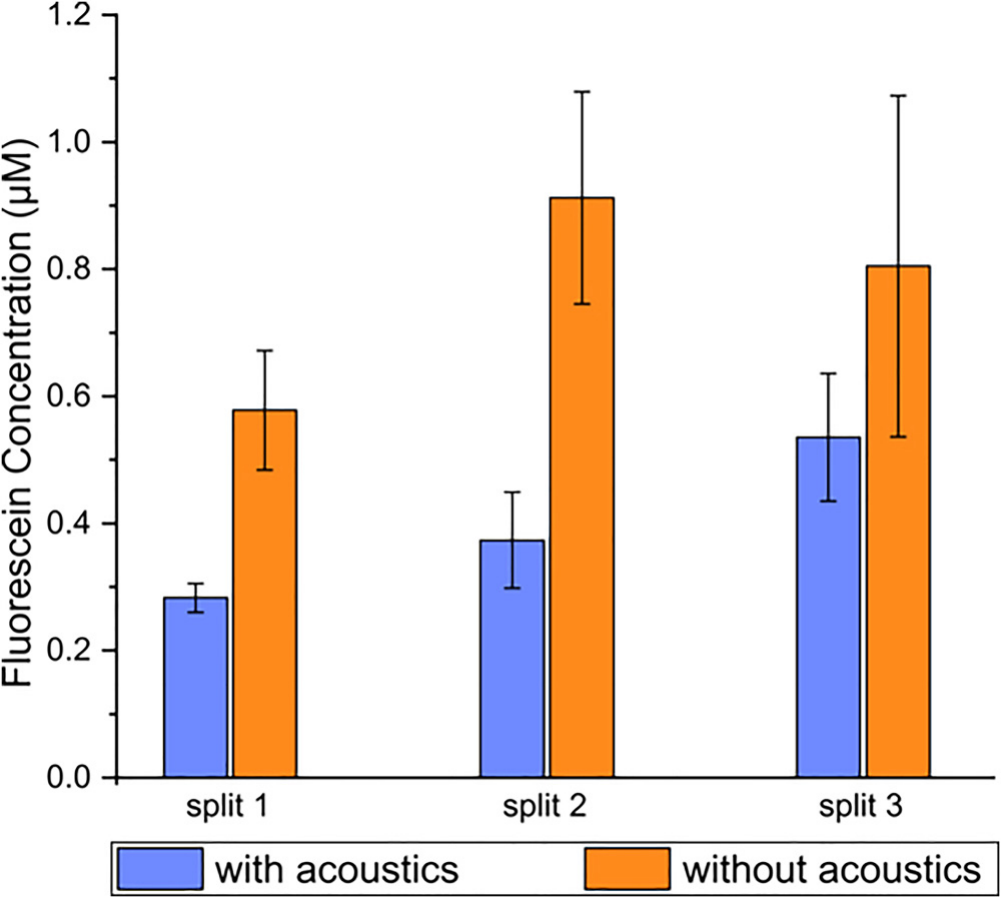
Fluorescein concentration in the side daughter droplets with or without acoustics actuated in the main channel during the droplet splits. When no acoustics was applied, the enzymes were not separated from the droplets meaning that the enzymatic reaction continued also off-chip.

To demonstrate the ability to stop the enzymatic reaction by separating the enzyme-coupled microbeads from the droplets, parts of the droplets were separated into the side outlet channel with and without acoustics and transferred to a PDMS chip where the fluorescence intensity was measured. The microbeads were focused in the main channel by the acoustics and, thus, concentrated in the droplets continuing in the main channel, Fig. 1(a). The fluorescence intensity of the droplets exiting the side outlet channel was dependent on the reaction time, i.e., the distance between the pico-injector and the split (Fig. 3). The droplets in split 3 displayed a higher fluorescence signal than the droplets at split 1 and split 2, as expected. When the acoustics was not applied, the enzyme-coupled microbeads continued to catalyze the FDP conversion in the droplets in the side outlet channels, and, therefore, the intensity was higher without acoustic actuation than with the acoustics applied (Fig. 3). From these data, we can see that R^2^ (coefficient of determination) with acoustic actuation is 0.97, indicating that the increase in fluorescence intensity is linear with the reaction time. For the data without acoustic actuation, R^2^ is only 0.46, thus showing an irregular variation. When the acoustics was applied, the fluorescence intensity was increased at the three splits as the reaction time could be controlled by the channel length. Without the acoustics, the fluorescence concentration varied irregularly as the reaction time was not possible to control.

**FIG. 4.**
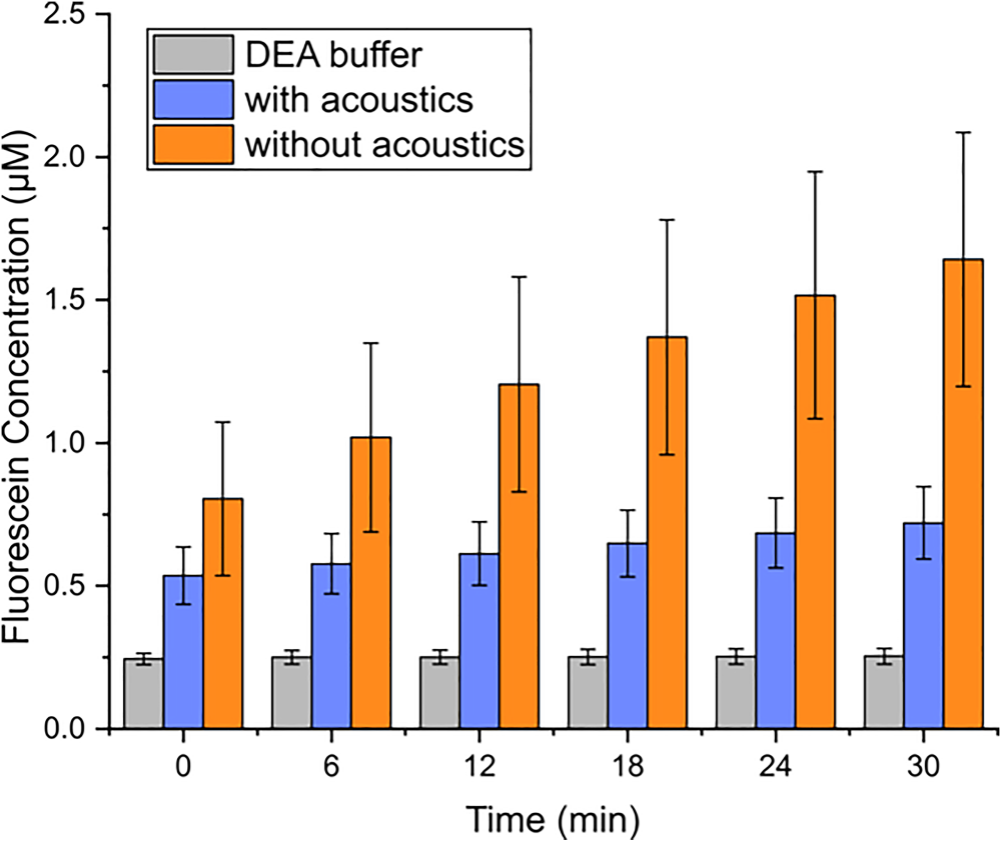
Fluorescein concentration in droplets collected at split 3 with or without acoustics applied. Data show how the concentration varied over 30 min. (For the DEA buffer, R^2^ = 0.843; for the FDP with acoustics, R^2^ = 0.999; for the FDP without acoustics, R^2^ = 0.991.)

To evaluate the ability of this platform to stop the enzymatic conversion reactions, 10 *μ*M FDP was used to generate droplets and the bead solution was injected into the droplets via the pico-injector. Fluorescent intensity of the collected droplets from split 3 with acoustics or without acoustics applied was followed for 30 min at detection intervals of 6 min. The DEA buffers were injected into droplets containing FDP through the pico-injector as controls, Fig. 4. The fluorescein concentration in the droplets without acoustics was increasing drastically (from 0.80 to 1.54 *μ*M) as expected when the enzymatic conversion reaction could continue off-chip. For comparison, the fluorescein concentration in the droplets collected with acoustics was increasing only slightly (from 0.53 to 0.72 *μ*M) during the 30 min time interval. The slight increase in the intensity even with the acoustics applied could be caused by detached enzymes from the beads or a few unfocused beads that were withdrawn together with the side daughter droplet. Droplets containing FDP in a DEA buffer has a stable intensity readout (0.25 *μ*M), as expected. In summary, we demonstrate the ability to stop the chemical reaction by removing the enzyme-coupled beads and perform readout on the supernatant in the droplets, off-chip.

## V. CONCLUSIONS

We have developed a microfluidic platform suitable for time-controlled reactions in droplets without the need of chemical inhibitors and have demonstrated proof-of-concept by using the standard enzyme system ALP conversion of FDP. In our system, the reaction was started by the pico-injection of enzyme-coupled microbeads and was stopped by removing the enzyme-coupled microbeads using acoustophoresis. We demonstrate that the reaction time can be controlled by using different outlets of the microfluidic reaction channel and that this does not affect the size of the generated droplets, volume injected via the pico-injector, nor the size of droplet that could be taken off-chip for analysis. The novelty in this work is the demonstration of a platform where the analysis can be performed off-chip to allow for longer exposure times compared to performing the analysis in-line. We believe that the platform could be a useful tool to determine the reaction kinetics of many different types of chemical reactions. The system is not limited to fluorescence detection of the product but also has the possibility to be integrated with other measurement techniques that can be performed off-chip.

## Data Availability

The data that support the findings of this study are available from the corresponding author upon reasonable request.
